# Less Is More: Psychologists Can Learn More by Studying Fewer People

**DOI:** 10.3389/fpsyg.2016.00934

**Published:** 2016-06-17

**Authors:** Matthew P. Normand

**Affiliations:** Department of Psychology, University of the PacificStockton, CA, USA

**Keywords:** experimental design, idiographic research, research methods, single-case designs, inferential statistics

Psychology has been embroiled in a professional crisis as of late. The research methods commonly used by psychologists, especially the statistical analyses used to analyze experimental data, are under scrutiny. The lack of reproducible research findings in psychology and the paucity of published studies attempting to replicate psychology studies have been widely reported (e.g., Pashler and Wagenmakers, 2012; Ioannidis et al., 2014; Open Science Collaboration, 2015). Although it is encouraging that people are aware of problems evident in mainstream psychology research and taking actions to correct them (e.g., Open Science Collaboration, 2015), one problem has received little or no attention: the reliance on between-subjects research designs. The reliance on group comparisons is arguably the most fundamental problem at hand because such designs are what often necessitate the kinds of statistical analyses that have led to psychology's professional crisis (Sidman, 1960; Michael, 1974; Parsonson and Baer, 1978). But there is an alternative.

Single-case designs involve the intensive study of individual subjects using repeated measures of performance, with each subject exposed to the independent variable(s) and each subject serving as their own control (Sidman, 1960; Barlow et al., 2008; Johnston and Pennypacker, 2009; Kazdin, 2010). Comparisons of performance under baseline and experimental conditions are made for each subject, with any experimental effects replicated with the individual subject across time or across multiple subjects in the same experiment. Single-case experiments yield data that can be interpreted using non-inferential statistics and visual analysis of graphed data, a strategy characteristic of other natural sciences (Best et al., 2001). Single-case experimental designs are advantageous because they more readily permit the intensive investigation of each subject and they achieve replication within an experiment rather than across experiments. Thus, data from just a few subjects tells a story.

## The importance of repeated measures

Psychologists tend to view the population of interest to be people, with the number of individuals studied taking precedent over the extent to which each individual is studied. Unfortunately, studying large groups of people makes repeated measurement of any one person difficult. The consequence is that we often end up knowing very little about very many.

**Figure 1:**
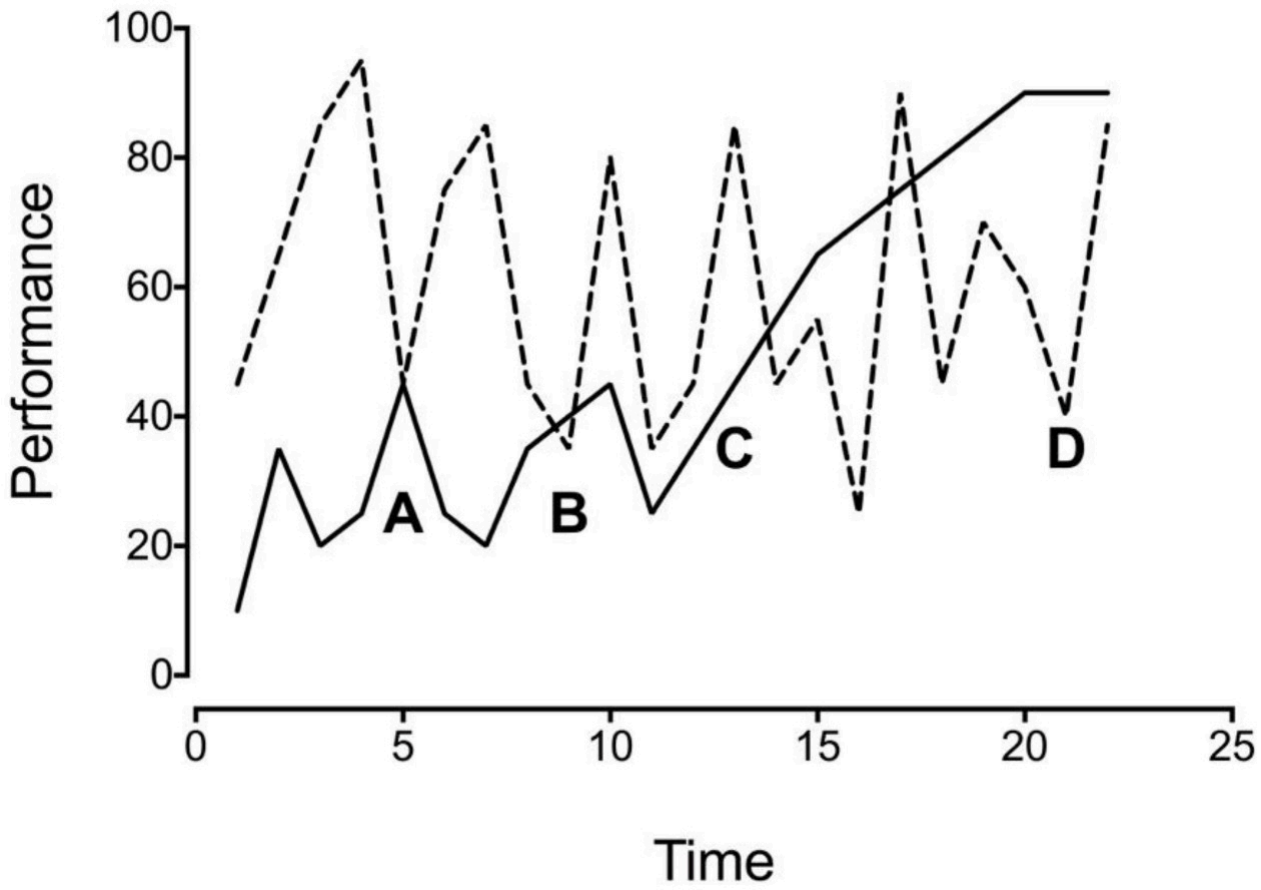
**Hypothetical data of repeated performance measures for two subjects**.

Instead, repeated measures of an individual's performance should constitute the relevant “population”—a population of representative individual performance measures. For internal validity, having representative samples of performances is more important than having a representative sample of a population. When you have only one or a few measures of each individual's performance, it is impossible to know how representative those measures are for the individual, never mind the population. Consider Figure 1, which depicts hypothetical data from two subjects. If you sampled the two performances at points A and B, you would conclude that they were similar. However, if you sampled the performances at points C and D, you would conclude that they were quite different. You can see from the complete data set, however, that neither sampling accurately reflects the performance of either subject. If the data do not generalize even to the individual, they are unlikely to generalize to the population as a whole.

## The actual vs. the average

The degree to which you understand a phenomenon is proportional to the degree to which you can predict and, where possible, control its occurrence. This is not simply a matter of showing that, on average, a certain outcome is more likely under a certain condition, which is what most of psychology research shows (Schlinger, 2004). Knowing that the performances of all subjects in an experiment averaged to a certain value does not predict the performance of any individual subject, except in a probabilistic way. This is a problem for the basic researcher trying to discern general laws or theories, and it is a problem for the practitioner who needs to help the individual (Morgan and Morgan, 2001). As Skinner quipped, “No one goes to the circus to see the average dog jump through a hoop significantly oftener than untrained dogs” (Skinner, 1956, p. 228).

More recently, Barlow and Nock (2009) asserted, “whether it's a laboratory rat or a patient in the clinic with a psychological disorder, it is the individual organism that is the principle unit of analysis in the science of psychology” (p. 19). Individuals behave, not averages. People don't respond “on average,” they respond a certain way at a certain time. This is no matter of opinion, it has to be true. The average is a statistical construct, derived from two or more performances, not a feature of the natural world (Sidman, 1952, 1960). Further, the statistical relation between some value of the independent variable and an average value of the dependent variable is not a real relation. The problem is made worse when the independent variable varies from case to case, as, for example, with psychotherapy procedures. You then are dealing with a statistical relation between an average value of an independent variable and an average value of a dependent variable. Relying on averaged performances also means that you have no effect without the data from all of your subjects because the effect never occurred independent of the statistical analysis.

Returning to Figure 1, note that the two subjects performed differently over time. As such, it is not simply a matter of collecting samples of performance from many different people to smooth the rough edges. You can average the two performances, but the result will not accurately describe either. Repeating this many times across many subjects does not improve the situation.

It also does no good to average the performance of an individual, as doing so obscures the variability evident in the individual performance. Averaging either subject's data from Figure 1 would obscure important features of those data. In one case, it would obscure a cyclical pattern of performance. In the other case, it would obscure an increasing trend across time. Variability is something to be understood, not ignored. To average it away is to assume that it is unimportant because it does not represent the real world. But variability does not obscure the real world, it is the real world. There is an important difference between controlling variability and “controlling for” variability. Controlling variability is a matter of experimental technique, whereas controlling for variability is a matter of statistical inference. Contrary to the way they are typically used, averages are most appropriate when the data in question are fairly stable. To quote Claude Bernard, the father of modern experimental medicine:

[W]e must never make average descriptions of experiments, because the true relations of phenomena disappear in the average; when dealing with complex and variable experiments, we must study their various circumstances…averages must therefore be rejected, because they confuse, while aiming to unify, and distort while aiming to simplify. Averages are applicable only to reducing very slightly varying numerical data about clearly defined and absolutely simple cases (Bernard, 1865/1957, p. 135).

In single-case experiments, the focus is on repeated measures of individual performance, not the average performance, with experimental control demonstrated subject-by-subject. If performances are stable and similar, then averaging the data can be a useful way to summarize the results. If not, averaging performances will obscure relevant functional relations or suggest functional relations where none exist.

## Replication and generality

Replication is the focal issue of psychology's current public relations crisis (Open Science Collaboration, 2015), as between-subjects experiments that rely on null-hypothesis testing and statistical significance can only be interpreted in the context of multiple replications. Knowing that a single study of 500 people produced an experimental effect that was significant at the 0.05 level tells us relatively little about the likelihood that the effect was real. Unfortunately, many people believe that a *p*-value of 0.05 means either that there is only a 0.05 chance that there was no experimental effect, or that there is only a 0.95 chance that the results are replicable. Neither interpretation is correct.

A significance level of 0.05 means that you would expect to get the dataset in question 5 out of every 100 times if the null hypothesis is true. With a single study, it is quite possible that you produced one of those five datasets. The only way to reject a null hypothesis is to conduct multiple similar studies that produce similar results. Moreover, to reject a null hypothesis based on a low *p*-value requires reversing the direction of the conditional probability, which is a mathematical error (Branch, 2009). For example, if the probability of it being cloudy given that it is raining is 0.95, this does not mean that the probability of it raining given that it is cloudy is 0.05. A null hypothesis cannot be rejected on the basis of a single study, no matter the widely-held beliefs to the contrary.

Single-case research designs involve replication of the experimental effect within the experiment, either within the individual subjects or across the subjects in the same experiment. The degree of internal validity possible with single-case research provides the foundation for replications across subjects and settings. Replication is possible when the relevant variables are identified—similar effects will be produced under circumstances in which those variables are present. Repeated performances on some experimental task are measured during baseline periods when the independent variable is not present and compared to repeated measures of the same performance when the independent variable is present. Each time behavior changes systematically when the condition changes, the experimental effect is replicated. The more replications, the more convincing the demonstration of experimental control^1^.

Despite the advantages in terms of internal validity, some assume that findings from single-case designs have limited external validity because data obtained from a few subjects might not generalize to a population at large. Actually, single-case research is precisely the way to establish generality, because to do so one first has to identify the relevant controlling variables for the phenomenon under study (Sidman, 1960). Generality is best established inductively, moving from the single case to ever-larger collections of single cases experiments with high internal validity. To have external validity you must first have internal validity (Guala, 2003; Hogarth, 2005). Without a complete understanding of the relevant variables, it is difficult to specify the circumstances in which you are likely to produce a given effect. Thorngate (1986) put it this way: “To find out what people do in general, we must first discover what each person does in particular, then determine what, if anything, these particulars have in common…” (p. 75).

Between-subjects designs are sometimes appropriate for what might be referred to as “engineering problems” (Sidman, 1960). For example, determining the effect a psychological intervention is likely to have in a large-scale delivery under naturalistic conditions. However, this is an endpoint along the research continuum. The path running from the establishment of internal validity to the demonstration of external validity is long and sometimes winding, but that is how science progresses. Leaping ahead means you miss some important landmarks along the way.

## Conclusion

Single-case research designs enjoy both history and currency in the natural sciences. In psychology, such designs have a storied history (Boring, 1929) but are currently out of favor (Morgan and Morgan, 2001; Barlow et al., 2008; Barlow and Nock, 2009). This is not necessarily for the better. Although between-subjects experiments certainly have their place, psychology would benefit if more researchers studied fewer subjects, took repeated measures of the subjects they study, and established generality inductively and systematically across individual subjects before turning to between-subjects research. All of these are reasons for emphasizing single-case research, and psychology will advance quicker and farther as a natural science and produce more effective technologies if it does. Size does matter. Sometimes, less is more, and we can learn more by studying fewer people.

## Author contributions

The author confirms being the sole contributor of this work and approved it for publication.

### Conflict of interest statement

The author declares that the research was conducted in the absence of any commercial or financial relationships that could be construed as a potential conflict of interest.

## Footnotes

^1^A number of other single-case designs have been developed to address different experimental manipulations and different dependent variables, and there are many comprehensive reviews of single-case experimental designs available (e.g., Barlow et al., 2008; Johnston and Pennypacker, 2009; Kazdin, 2010).
